# Secure corridor for infraacetabular screws in acetabular fracture fixation—a 3-D radiomorphometric analysis of 124 pelvic CT datasets

**DOI:** 10.1186/s13018-018-0833-y

**Published:** 2018-05-21

**Authors:** Stephan Arlt, Hansrudi Noser, Andreas Wienke, Florian Radetzki, Gunther Olaf Hofmann, Thomas Mendel

**Affiliations:** 1 Department of Trauma Surgery, BG Klinikum Bergmannstrost Halle gGmbH, Merseburger Straße 165, 06112 Halle (Saale), Germany; 2 Department of Trauma Surgery, Univeritätsklinikum Jena, Am Klinikum 1, 07747 Jena, Germany; 3 AO Research Institute, Clavadelerstrasse 8, CH-7270 Davos Platz, Switzerland; 4 Martin Luther University Halle-Wittenberg, Institute of Medical Epidemiology, Biometry and Informatics, Magdeburger Str. 8, 06112 Halle (Saale), Germany; 5 Department of Orthopaedic and Trauma Surgery, Martin Luther University Halle-Wittenberg, Ernst-Grube-Straße 40, 06120 Halle (Saale), Germany

**Keywords:** Infraacetabular screw, Acetabulum, Acetabular fracture, Virtual bone corridor, Corridor volume, Computed tomography, Computer-aided imaging

## Abstract

**Background:**

Acetabular fracture surgery is directed toward anatomical reduction and stable fixation to allow for the early functional rehabilitation of an injured hip joint. Recent biomechanical investigations have shown the superiority of using an additional screw in the infraacetabular (IA) region, thereby transfixing the separated columns to strengthen the construct by closing the periacetabular fixation frame. However, the inter-individual existence and variance concerning secure IA screw corridors are poorly understood.

**Methods:**

This computer-aided 3-D radiomorphometric study examined 124 CT Digital Imaging and Communications in Medicine (DICOM) datasets of intact human pelves (248 acetabula) to visualize the spatial IA corridors as the sum of all intraosseous screw positions. DICOM files were pre-processed using the Amira® 4.2 visualization software. Final corridor computation was accomplished using a custom-made software algorithm. The volumetric measurement data of each corridor were calculated for further statistical analyses. Correlations between the volumetric values and the biometric data were investigated. Furthermore, the influence of hip dysplasia on the IA corridor configuration was analyzed.

**Results:**

The IA corridors consistently showed a double-cone shape with the isthmus located at the acetabular fovea. In 97% of male and 91% of female acetabula, a corridor for a 3.5-mm screw could be found. The number of IA corridors was significantly lower in females for screw diameters ≥ 4.5 mm. The mean 3.5-mm screw corridor volume was 16 cm^3^ in males and 9.2 cm^3^ in female pelves. Corridor volumes were significantly positively correlated with body height and weight and with the diameter of Köhler’s teardrop on standard AP pelvic X-rays. No correlation was observed between hip dysplasia and the IA corridor extent.

**Conclusion:**

IA corridors are consistently smaller in females. However, 3.5-mm small fragment screws may still be used as the standard implant because sex-specific differences are significant only with screw diameters ≥ 4.5 mm. Congenital hip dysplasia does not affect secure IA screw insertion. The described method allows 3-D shape analyses with highly reliable results. The visualization of secure IA corridors may support the spatial awareness of surgeons. Volumetric data allow the reliable assessment of individual IA corridors using standard AP X-ray views, which aids preoperative planning.

## Background

Since the prodigious surgical oeuvre of Letournel and Judet in the 1960s, the surgical fixation principles for the treatment of displaced acetabular fractures associated with instability or incongruency of the hip joint have remained mainly unchanged [1, 2]. Thereby, accurate anatomical joint reconstruction mostly by open reduction and internal fixation (ORIF) is still considered to be the key factor for an optimal clinical outcome and for reducing the risk of posttraumatic osteoarthritis [3–8]. Depending on the fracture type, numerous stabilization methods have been developed in the past several decades that allow early patient mobilization to prevent complications such as venous thromboembolism, pneumonia, or muscular atrophy. All fixation constructs attempt to stabilize the alignment of the fractured anterior or posterior (or both) acetabular column(s).

Due to demographic changes, the incidence of acetabular fractures in older adults has steadily increased in recent decades, a trend that will inevitably continue in the coming years [9–12]. Operative treatment of these patients is more complex due to their higher rate of comorbidities, including lower immune defenses and, in particular, their osteoporotic bone stock. The latter makes adequate stabilization by plates and screws difficult because of the weak implant-bone interface. Additionally, the inability of elderly patients to unload the affected leg postoperatively places greater mechanical strain on the fixation construct, often leading to secondary implant loosening and fracture reduction loss. Particularly in fracture patterns with a separation of both columns through the acetabulum and extending into the obturator foramen, distension of the specifically fixed load-bearing columns, which leads to central protrusion of the femoral head, must be avoided. Such fracture patterns include T-shaped fractures (62-B2), both column fractures (62-C), posterior column fractures (62-A2), and anterior column posterior hemi-transverse fractures (62-B3).

**Fig. 1:**
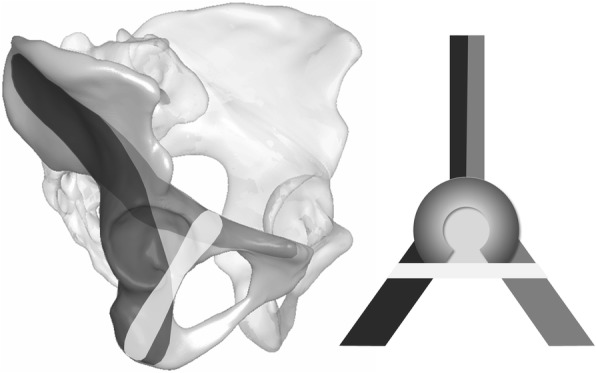
Infraacetabular screw track for the completion of a periacetabular fixation frame interconnecting the separated anterior and posterior columns

To address this lack of fixation strength, an additional construct is desirable that would transfix the anterior and posterior columns beneath the acetabular fossa to close the periacetabular fixation frame [13] (Fig. 1). Emile Letournel cited a screw insertion parallel to the quadrilateral wall and medial to the joint in his well-known book, “Fractures of the Acetabulum” [14]. However, this bone zone is quite small. Hence, in the case of malpositioning, the screw insertion places the adjacent inner pelvic structures and the hip joint itself at potential risk. Currently, the spatial proportions of the infraacetabular (IA) bone region corresponding to the thin bone mass of the quadrilateral surface at the level of the acetabular fossa projecting to Köhler’s teardrop on the anteroposterior (AP) pelvic X-ray view are poorly understood.

Furthermore, the proposed spatial shape of such a screw corridor and its structural anatomical limitations are lacking, which explains why the IA screw has not gained wide acceptance in the acetabular trauma surgeon community. Reports have rarely described the routine application of an IA screw for conventional osteosynthesis of the impaired column(s) [15].

However, recent biomechanical investigations have clearly demonstrated the superiority of a preferably inferiorly positioned interconnecting screw in the IA region beneath the acetabular fossa [13, 16]. Gras et al. [17] recently presented a mean-shape analysis of 523 healthy pelves and found the existence of an IAC with a minimum corridor diameter of 5 mm in 93%.

In our study, a computer-assisted 3-D radiomorphometric analysis of the IA region was used to assess the presence, true shape, and extent of the intraosseous secure screw corridor. This analysis was performed based on conventional CT Digital Imaging and Communications in Medicine (DICOM) datasets using custom-made software algorithms and differs from Letournel’s original description of the possible screw positions along the quadrilateral wall by allowing the partial penetration of the acetabular fossa without altering the cartilage of the femoral head [14].

Furthermore, a possible relationship between the IA corridor dimensions and the presence of dysplastic acetabular shape variants warrants investigation. In this regard, some authors have reported the widening of the quadrilateral plate due to a traumatic lesion of the triradiate cartilage in infantile acetabula, resulting in posttraumatic dysplasia [18, 19]. Consequently, a lateralization of the hip’s center of rotation accompanied by lesser roofing of the femoral head may be hypothesized to result in a wider bone stock of the quadrilateral plate at the level of the acetabular fovea, even in cases of congenital hip dysplasia.

## Methods

### Epidemiology and data pre-processing

For this study, 124 CT datasets of unimpaired human pelves from a Caucasian cohort provided the basis for the virtual analysis of the existence, shape, and variability of the IA corridor. The datasets were primarily prepared by the diagnostic radiology department of the Martin Luther University of Halle-Wittenberg, Germany, for the diagnosis of individual diseases from September 2008 to August 2010. All patients declared their consent for further scientific use of their anonymized imaging data. The CT scans were generated by a SOMATOM Sensation 64 Multislice CT scanner (Siemens AG, Erlangen, Germany). The image resolution was 512 × 512 pixels, the slice distance was 0.4 mm, and the slice thickness was 0.6 mm.

The generated DICOM files were pre-processed semiautomatically by a custom-made C++ software algorithm implemented in the scientific visualization software Amira® (Visage Imaging, Berlin, Germany). The first preparatory steps included the anonymization, cropping, labeling, and segmentation of the datasets. Next, gray-scale values of the voxels were labeled with a value of “1” if representing bone. Voxels on the exterior of the pelvic bone were labeled with a value of “0”, and the voxels comprised an STL data file. This process represented a key step in transforming the 3-D anatomical shape conditions of the pelvis into a valuable matrix for a mathematical software algorithm. Details of this standardized workflow were described in previously published studies by our working group [20–25].

### Spatial alignment

Twelve anatomical landmarks were set using a mouse-click on the bone surface of each 3-D pelvic model to allow spatial alignment in the standard Matta “inlet” and “outlet” views. To compute the IA corridor, the establishment of three supplemental landmarks was required. For this purpose, the corridor was adjusted in an orthograde manner in a semi-transparent display mode to set the first landmark (P0) on the bony surface in the center of Köhler’s teardrop in the region of the iliopubic eminence. The second landmark (P1) was set on the projection in the region of the ischial tuberosity. The conjunction of these entry and exit points represented the basic valid vector (BVV) for an IA screw track entirely situated in the compact bone, with a voxel transition sequence of “0-1-0” signifying “no bone–bone–no bone”.

### Operating principles of the IA corridor algorithm

**Fig. 2:**
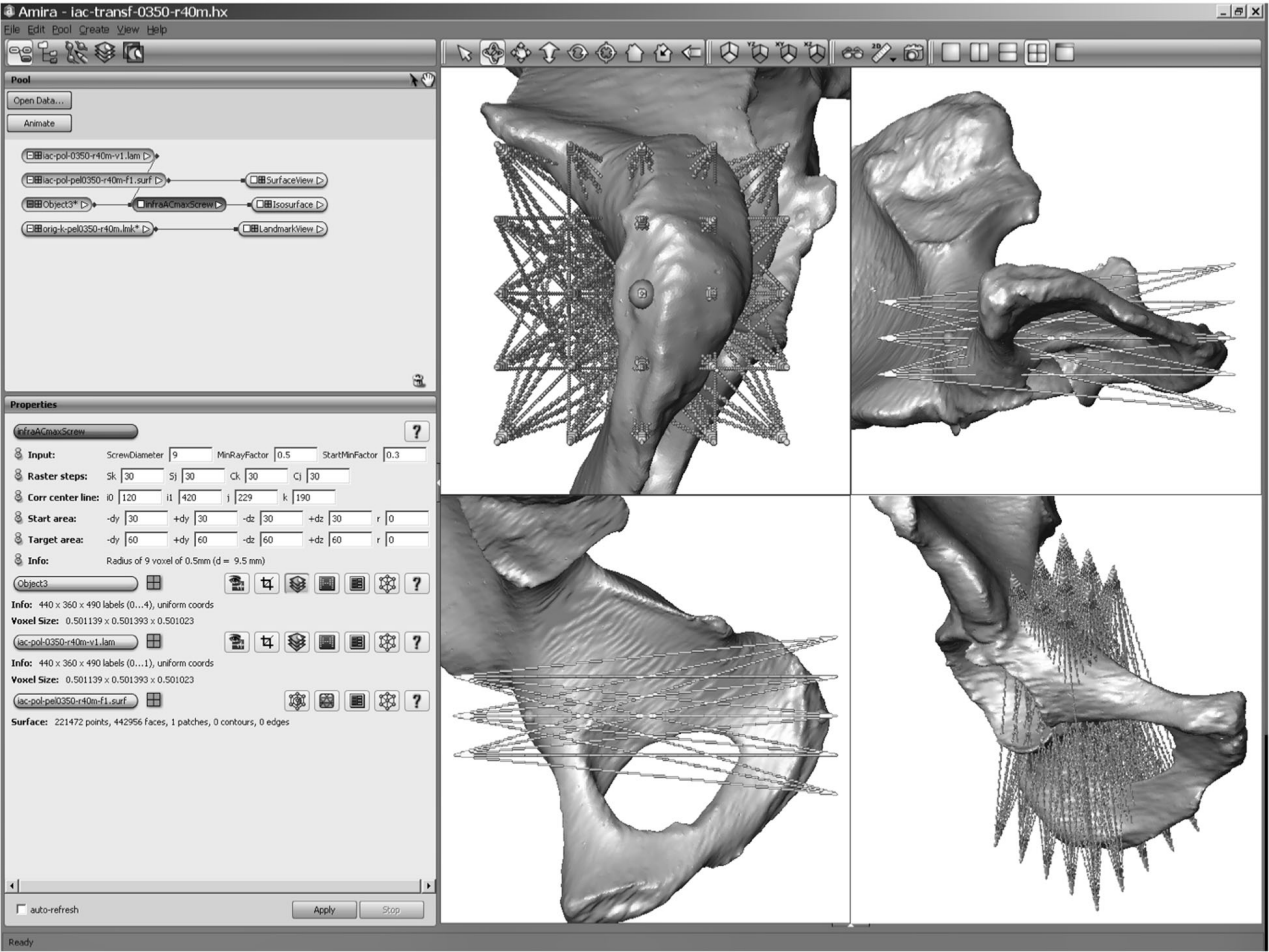
Bresenham lines generated from the start to the target area

A rectangular start and a target area were generated at a distance of 50 voxels above P0 and 15 voxels beneath P1 using another custom-made software module. The intersecting BVV corresponded to the center point of each area. Subsequently, the transition sequences from every raster point of the start area and ending on the given raster points of a target area were registered by the script based on the Bresenham algorithm [26] (Fig. 2).

**Fig. 3:**
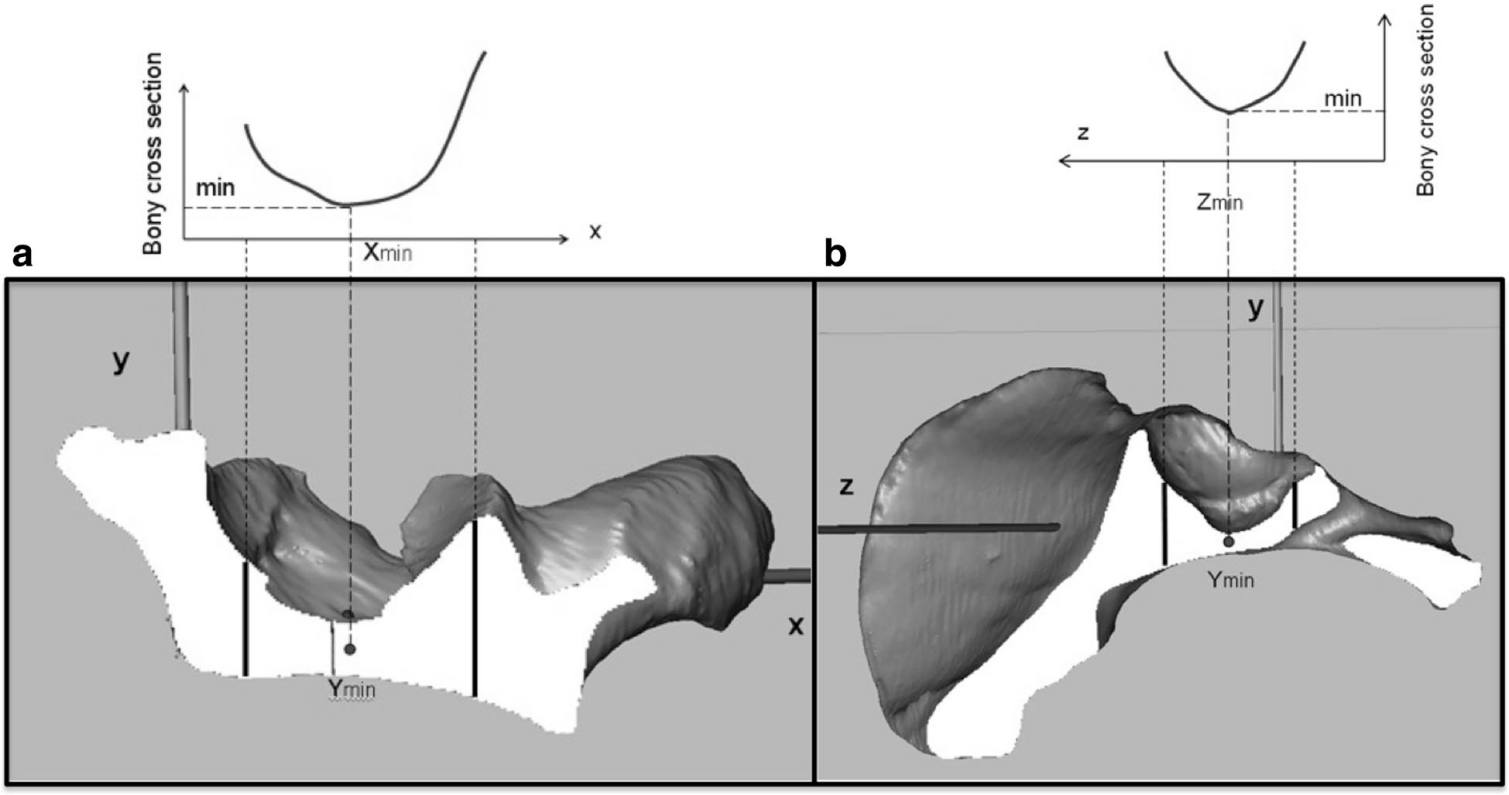
Script-based iterative isthmus search operation of PF along the *x*-axis (**a**) and *z*-axis (**b**)

All lines generating the transition sequence 0-1-0 were registered as potentially valid screw tracks. Higher sequences (e.g., 0-1-0-1-0) represented breaks beyond the bone during the course through the IA region and were therefore excluded by the script. Because they were not surgically pertinent, potential entry points medial to the pelvic brim with screw tracks passing through the IA bone region were avoided. For this reason, 5 to 10 landmarks were set by mouse-clicks along the terminal line for each investigated acetabulum. Bresenham lines that passed the bone medial to these landmarks were excluded automatically. Furthermore, screw tracks along the quadrilateral bone stock beyond the fovea acetabuli throughout the posterior column were avoided by an iterative search operation of the program script that determined the thinnest point (P_F_) in the acetabular fossa as the cut-off (Fig. 3). The segmentation of the entirety of all the valid screw tracks constituted the secure IA bone corridor. At the initial run of the script, the Bresenham lines had a thickness of 0.5 mm (1 voxel). By enlarging the line diameter in 1-mm intervals, the spatial IA corridor volumes could be generated for the different screw diameters commonly used in surgical fracture fixation. As a final operation, the script computed the volumetric values of the 3-D corridor models, namely, the volume and the isthmus, entry, and exit areas for further statistical analyses.

### 2-D anatomical analysis of planar pelvic projections

To determine whether the extension of the IA bone mass correlated with the dysplastic acetabular shape variance, correlations between the script-based volumetric corridor data and the commonly accepted radiological indices for hip dysplasia on planar pelvic X-rays were investigated. For this purpose, Wiberg’s lateral center-edge angle (LCE), Lequesne’s acetabular index (AI), and the Heyman and Herndon (DWI) depth-to-width index were assessed in the AP planar projection of each pelvis bilaterally [27]. Additionally, the diameter of Köhler’s teardrop in the AP pelvic view was measured for each acetabulum.

### Statistics

Statistical analysis was completed using SPSS 21® (SPSS Inc., Chicago, IL, USA). A confidence interval of 95% was assumed (significance level *p* < 0.05). The chi-square test was used to compare the gender-related corridor prevalence to the different screw diameters. The independent samples *t* test was performed to analyze sex- and laterality-related differences of the IA corridors. To allow predictive statements, multivariate regression analysis was performed to determine the influence of individual variables on the corridor extent. The descriptive statistics presented in this study include the mean and the first standard deviation.

### Ethical review committee

The study was approved by the independent ethical committee of the medical council of Saxony-Anhalt, Germany, and confirmed under approval no. 63/17.

## Results

The 124 investigated CT scans (248 hemipelves (HP) in total) included 75 males and 49 females. The epidemiological data are shown in Table 1. Computational procedures for the IA corridor calculation were completely performed for each dataset. The existence of corridors was examined for different screw diameters starting with 3.5 mm and increasing in 1-mm intervals up to 7.5 mm.

**Table 1:** Epidemiological data of the patient population

	Males *n* = 75	Females *n* = 49
	Mean ± SD	Mean ± SD
Age (years)	58 ± 17.2	61 ± 12.6
Height (cm)	177 ± 7.6	165 ± 8.1
Weight (kg)	80 ± 14.5	71 ± 14.0

*SD* standard deviation

**Fig. 4:**
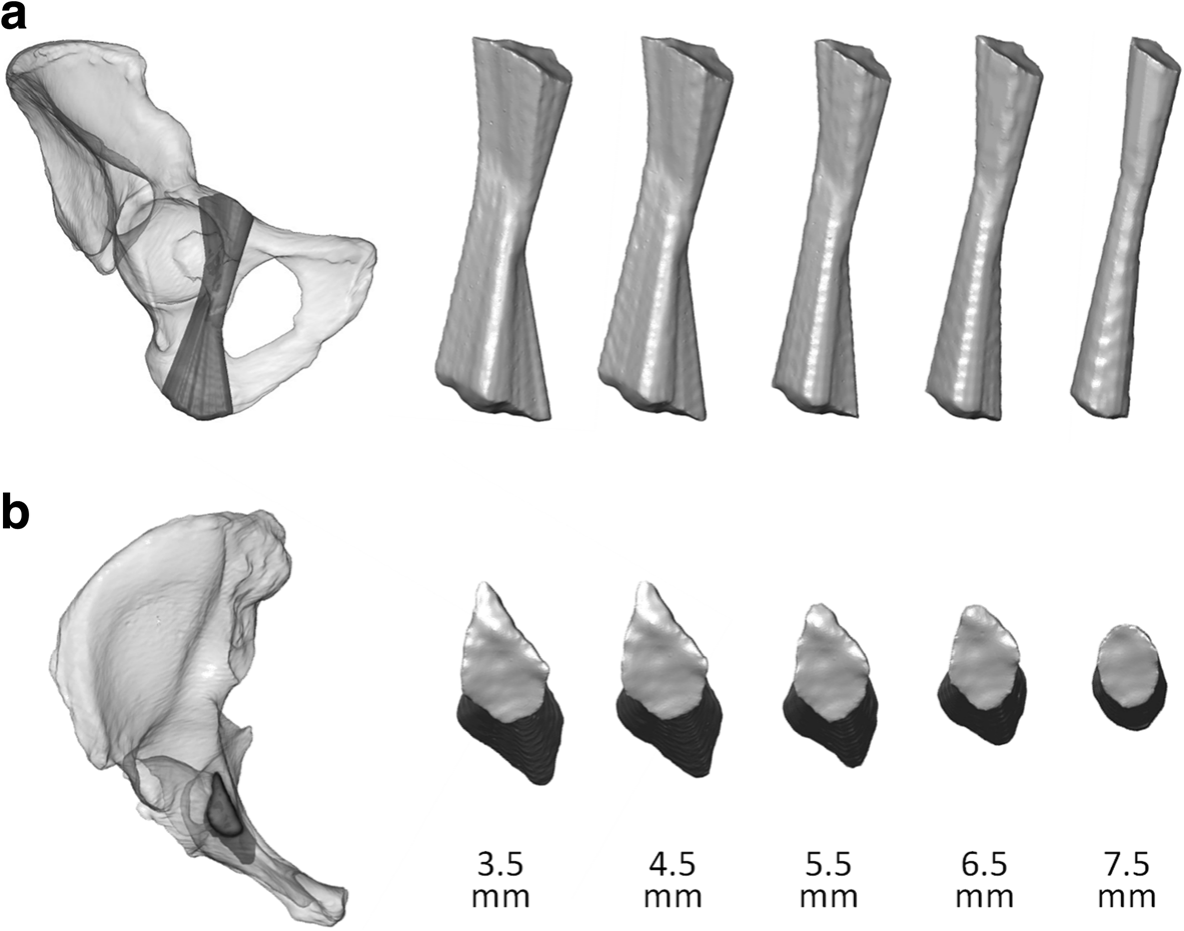
Morphological changes of the IA corridor caused by increasing screw diameters in **a** the obturator outlet view and **b** the inlet view

All IA corridor volumes consistently showed a double-cone shape with the isthmus located in the region of the acetabular fovea as the limiting anatomical structure. The screw entry areas were projected to the iliopectineal eminence. The smaller the screw diameter, the more the shape of the entry area conformed to a teardrop. With increasing screw diameters, the shape thus became more spherical, and the corridor volumes decreased to a tubular shape. The exit areas were consistently localized to the sciatic protuberance. The surface of both areas mutually decreased with higher screw diameters (Fig. 4).

**Fig. 5:**
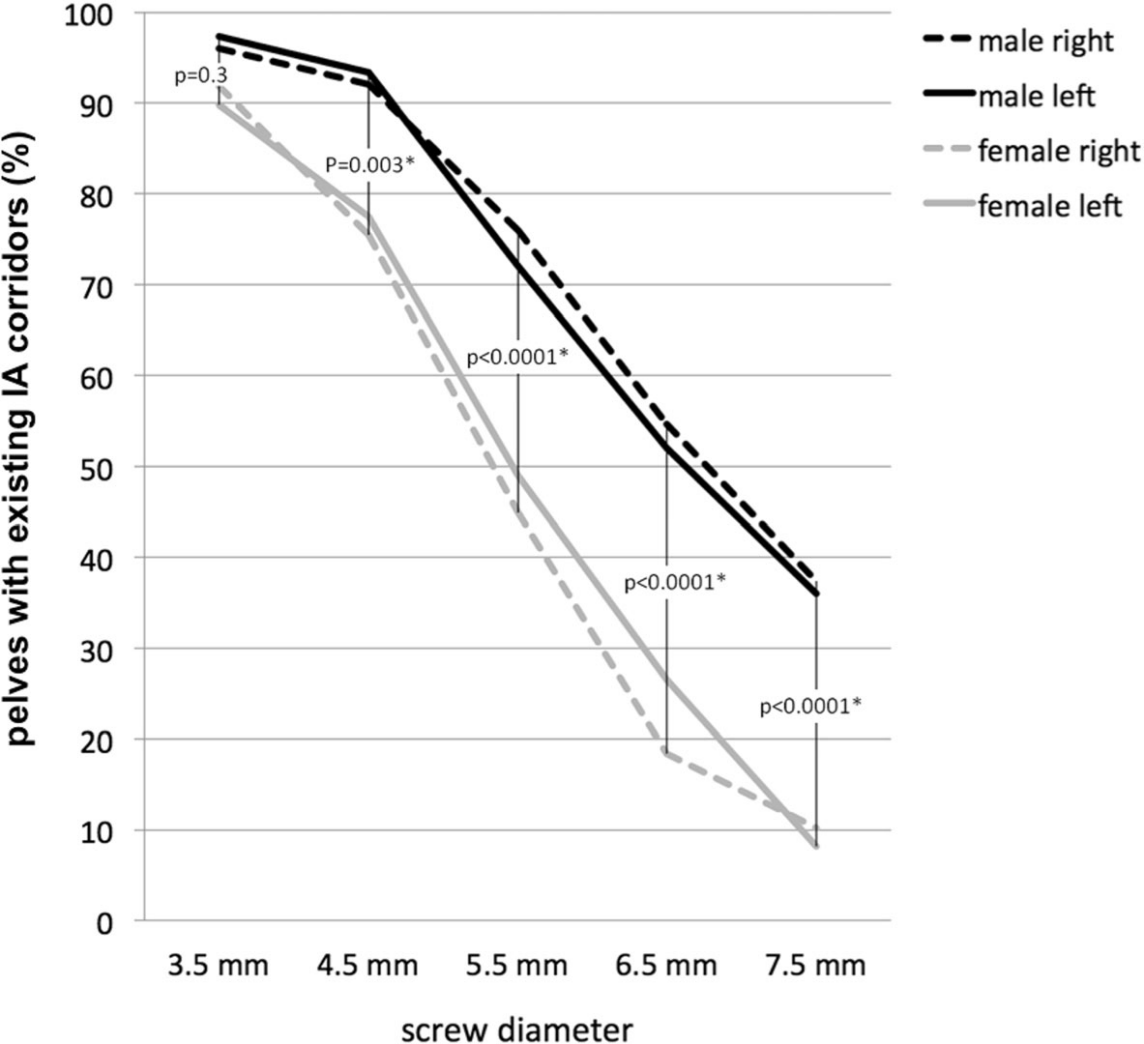
Gender-related existence of IA corridors for different screw diameters

In 145 of the 150 male HP (97%) and 89 of the 98 female HP (91%), the analysis revealed an existing corridor for a 3.5-mm screw. The chi-square test did not reveal any gender-related differences (*p* = 0.3). However, a safe IA bone path existed for a 4.5-mm screw in 139 male HP (93%), whereas only 75 female HP (77%) displayed sufficient bone space. This difference was highly significant (*p* = 0.003). This trend continued for screw diameters up to 7.5 mm, with significantly fewer IA corridors present in the female pelves than in the male pelves (*p* < 0.0001) (Fig. 5).

**Fig. 6:**
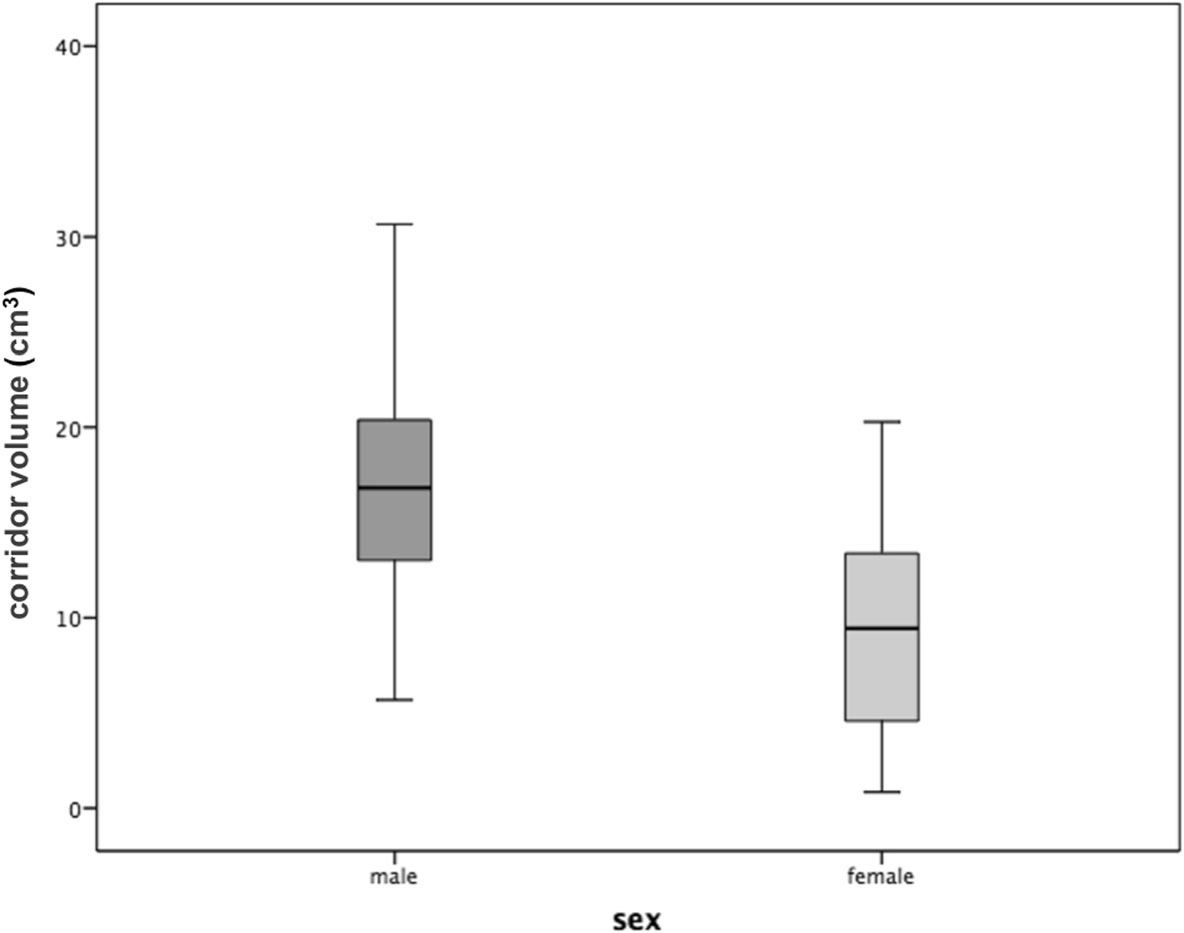
Sex-specific differences in the corridor volume

Based on these findings, we exclusively focused our further analyses on the 3.5-mm IA corridors, which existed in more than 90% of both genders and which is, therefore, the most appropriate implant diameter for this surgical application. However, although no difference in the corridor prevalence was observed for the 3.5-mm screws between genders, all the volumetric corridor data (namely, volume, entry and exit areas, and isthmus area) notably showed significant sex-specific differences (Table 2). With regard to the direct correlations between the shape and the proportion of the aforementioned corridor-describing parameters, the mean volume was the most useful and will, therefore, be considered for further applications. In this dimension, the mean corridor volume of the acetabula in males was 6.8 cm^3^ larger than that in females (*p* < 0.001) (Fig. 6).

**Table 2:** Volumetric data of 234 hemipelves with an existing 3.5-mm IA corridor

	Volume (cm^3^)		Entry area (mm^2^)		Exit area (mm^2^)		Isthmus area (mm^2^)	
	Male	Female	Male	Female	Male	Female	Male	Female
Mean	16	9.2	320	190	383	262	78	46
SD	5.7	4.6	127.5	97	126.5	122.1	32.1	24
Min	0.7	0.8	15.7	19.9	15	21	11.3	10.7
Max	32.8	20.2	627	437	862	554	210	108

*SD* standard deviation, *Min* minimum, *Max* maximum

**Fig. 7:**
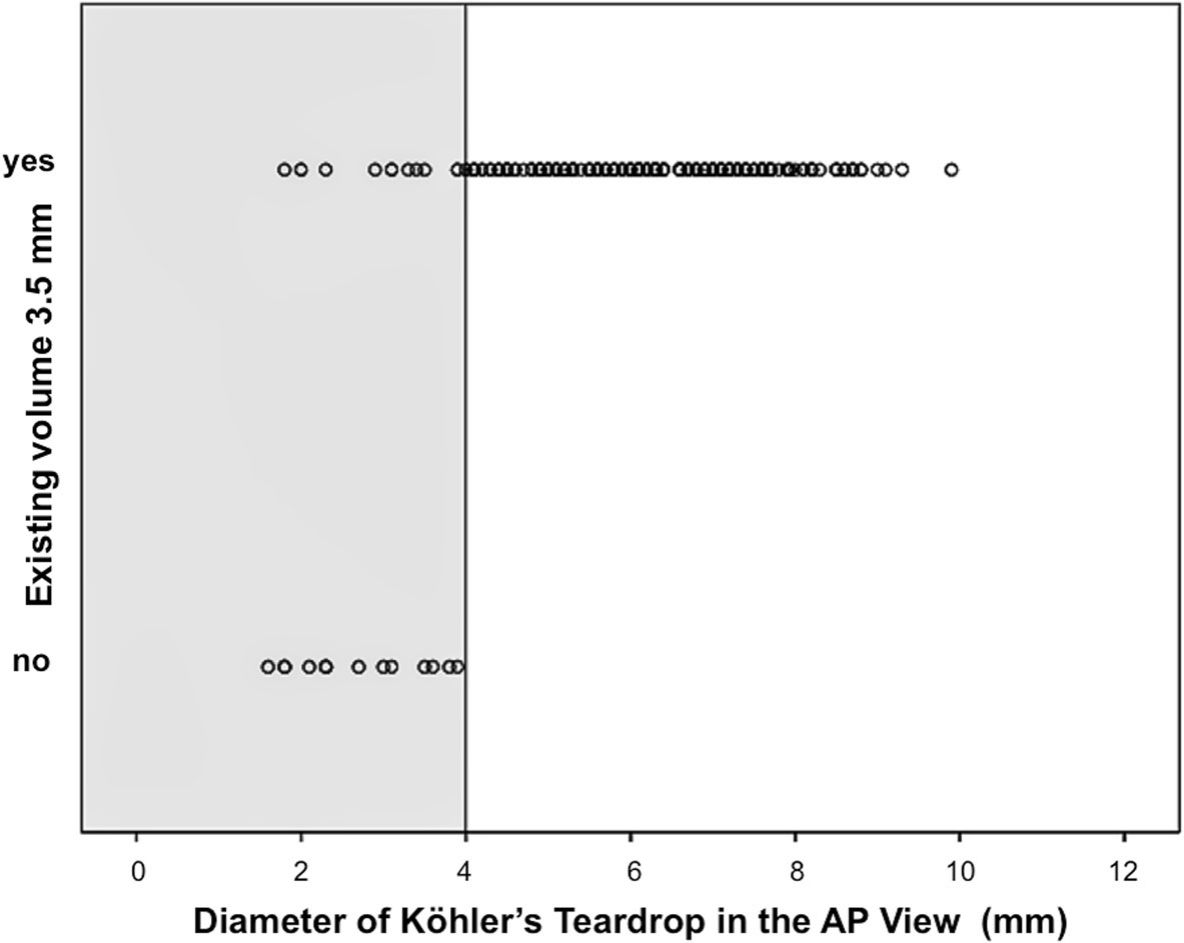
Corridor prediction and the diameter of Köhler’s teardrop measured in the AP pelvic view showing a 4-mm cut-off

Linear multivariate regression analysis was used to reveal the potential influence of the individual epidemiological data and the measurement values on the planar X-rays, which showed a reliable correlation with the extent of the corridor. In ascending sequence, the body weight (*r* = 0.3), body height (*r* = 0.4), and the diameter of Köhler’s teardrop in the AP X-ray view (*r* = 0.5) showed significant positive correlations with the corridor volume (coefficient of determination: *R*^2^ = 0.54; ANOVA *p* < 0.001). Therefore, Köhler’s teardrop diameter represented a strong predictive parameter for corridor existence in 100% of our cases, with a cut-off value of ≥ 4 mm (Fig. 7).

**Fig. 8:**
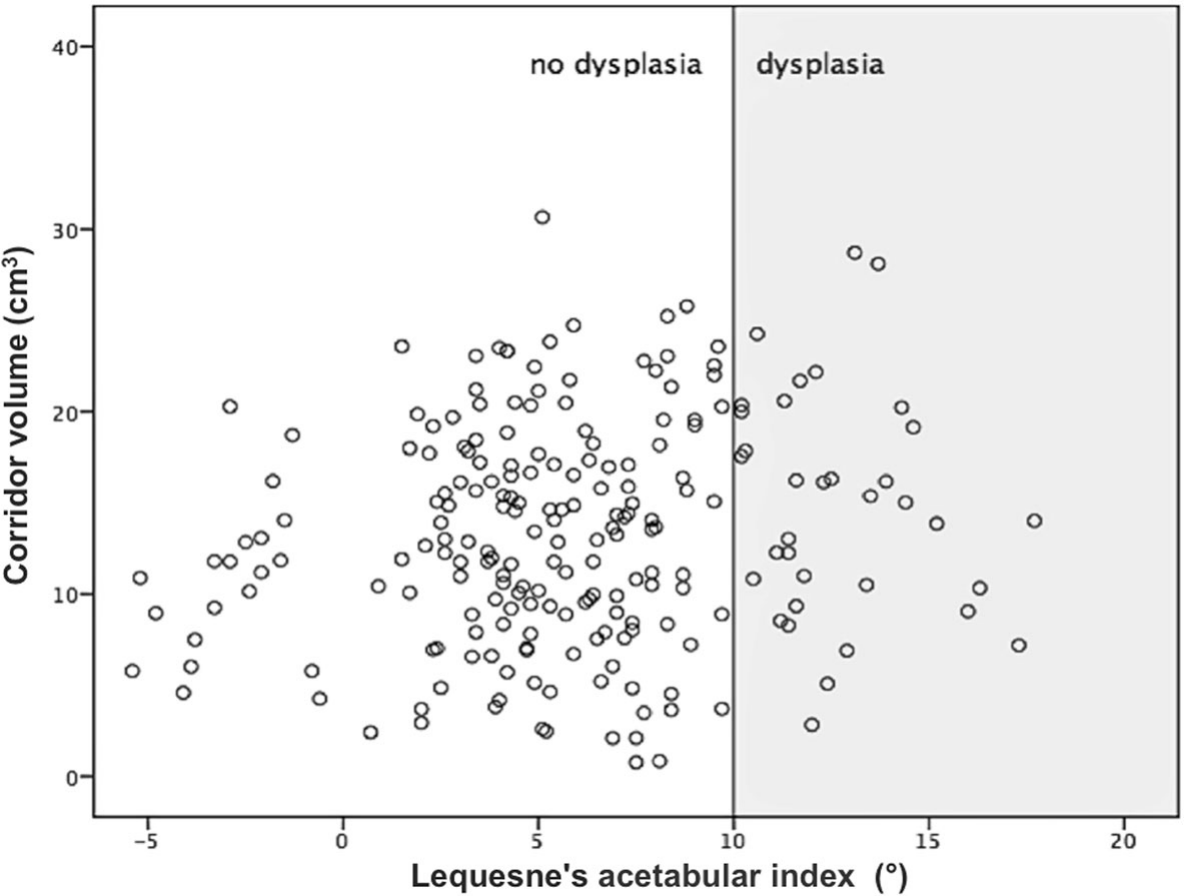
Scatter plot showing the distribution of the extent of dysplastic acetabular malformation and the IA corridor volume using the example of Lequesne’s acetabular index (AI)

According to our hypothesis of a lateralized rotational center and a lesser roofing of the femoral head leading to a wider bone stock of the quadrilateral plate at the level of the acetabular fovea in dysplastic hip shape variants, the following results were found. In the 248 hemipelves, we observed dysplastic malformations by measuring Wiberg’s lateral center-edge angle in 3 (2.4%), Lequesne’s acetabular index in 37 (14.9%), and the depth-to-width index according to Heyman and Herndon in 13 acetabula (5.2%) (Fig. 8). Multivariate regression analysis did not show any significant correlation between the corridor extent and the three aforementioned radiological indices for hip dysplasia (coefficient of determination: *R*^2^ = 0.02; ANOVA *p* = 0.2). Therefore, our hypothesis of increasing corridor volumes in cases of dysplastic hip variations could not be confirmed.

## Discussion

Acetabular surgery is one of the most challenging procedures in skeletal trauma. The complex bony anatomy and the difficult spatial orientation presented by this type of surgery contribute to this fact. Anatomical reduction and rigid fracture fixation are necessary to preserve hip joint function. Thus, numerous approaches and fixation techniques have been established over the past decades with the goal of restoring the integrity of the anterior and posterior columns. Additionally, recent biomechanical studies have shown that the fixation strength may be increased up to 50% for fracture patterns with a separation of both columns by closing the periacetabular fixation frame using an interconnecting IA screw [13, 16].

However, IA screw insertion is technically demanding and places adjacent neurovascular structures along with the hip joint at potential risk. The small isthmus at the level of the acetabular fovea requires accurate fracture reduction and precise screw insertion to avoid implant malpositioning. However, the small number of acetabular fracture cases [28] in routine clinical practice leads to a flat learning curve of surgeons, representing an additional barrier in this field [3]. To increase the safety of IA screw insertion, exact fluoroscopic imaging of the screw pathway is mandatory [17]. Otherwise, serious consequences such as iatrogenic lesions of vessels and nerves or penetration into the hip joint may result.

To date, the inter-individual variance of the secure infraacetabular bone path remains poorly understood. Therefore, a detailed understanding of the complex pelvic anatomy is of substantial surgical pertinence [17]. With this study of 248 hemipelves, we present a method to visualize the actual spatial shape of the individual osseous IA corridors based on precise 3-D CT reconstructions of all possible intraosseous screw positions crossing the quadrilateral section of the acetabulum. To achieve this, the IA corridors for different screw diameters commonly used in trauma surgery were computed by specially developed software algorithms. To the best of our knowledge, we are the first group to present a realistic visualization of this unfamiliar region of high biomechanical interest.

In accordance with the rapid developments of IT-based image processing, virtual anatomical research based on precise 3-D image data obtained from CT or MRI datasets has spurred the development of a wide range of new methods to answer specific questions. The obtained results may command both scientific [25, 29–31] and didactic interest [20, 24]. Furthermore, their role in preoperative surgical planning is expanding [17, 23, 32, 33]. The computational workflow needed to generate highly precise 3-D reconstructions from raw conventional pelvic CT datasets in this study represents a standardized procedure that has been published previously in several radiomorphometric studies [23, 25]. Moreover, our working group has investigated safe bone corridors in the sacroiliac region of the pelvis using specially developed software algorithms [21, 22].

In 2014, Gras et al. [17] reported the results of a biomorphometric CT-based analysis of 523 pelves regarding the IA corridor. They found a corridor diameter of at least 5 mm in 484 pelves (93%). Similar to our results, they found significantly smaller corridors in females than in males. However, their study focused on the spatial alignment of secure IA screw tracks in relation to different pelvic planes to allow for an improved intraoperative surgical orientation. They did not analyze the influence of increasing the screw diameter on the shape and spatial extent of the corridor. No volumetric analysis of the corridor volume was performed.

Our data showed that the shape, extent, and presence of a safe IA screw corridor differ according to certain individual variables. In both genders, an IA corridor for a 3.5-mm screw existed in more than 90% of all our cases. In females, however, the bone stock of the medial acetabular margin was consistently smaller (42% smaller than in males), which corresponded to the findings of Gras et al. [17]. Notwithstanding these observations, this difference is not germane to the frequency of existing corridors that may accommodate 3.5-mm screws, that is, 97 and 91% as observed herewith in males and females, respectively (*p* = 0.3). With increasing screw diameters of 4.5 mm or greater, however, the prevalence of a sufficient corridor significantly decreased in females (*p* < 0.05). Based on these findings, 3.5-mm small fragment screws should be used as the standard implant to transfix fracture separation of the anterior and posterior columns via the IA corridor.

Furthermore, the individual epidemiological variables of body height and weight were shown to strongly influence the existence and extent of the IA screw track. Both parameters showed significant positive correlations with increasing corridor volumes. Additionally, linear multivariate regression analyses of the volumetric corridor data revealed a significant correlation between the transverse diameter of Köhler’s teardrop on the standard AP pelvic X-ray view and a boundary value of ≥ 4 mm, allowing the secure insertion of a 3.5-mm screw. Therefore, we achieved the successful transfer of knowledge from virtual volumetric research to an easily measurable value that may serve as an important predictor in preoperative planning.

With regard to congenital hip dysplasia, our research did not focus on the joint pathology itself. In fact, it is important to ascertain whether hip dysplasia, which is easily identified on standard AP X-rays, may be used as a helpful indicator of the presence of a capacious IA bone corridor. However, our results disprove the hypothesis of a more capacious quadrilateral bone stock in hip dysplasia that would provide greater space for an IA screw. In contrast to observations in posttraumatic hip dysplasia after juvenile acetabular injury [18, 19], in congenital dysplasia, a lateralized rotational hip center with lesser roofing of the femoral head does not correlate with a widened IA region, thereby precluding a safer IA screw insertion.

## Conclusion

Our results provide a comprehensive understanding of the unique anatomic details of the quadrangular bone stock with regard to secure IA screw insertion. The described methods allow the computation of 3-D corridor volumes from conventional pelvic CT data implemented in the C++ computer language as a user-specific module of Amira® software [22]. The workflow allows the semiautomatic shape analyses of a large number of CT DICOM datasets with highly reliable results. The visualization of secure IA corridors may aid in training surgeons and in improving their spatial awareness.

The primary aim of this study was the determination of consistent anatomical landmarks that could regularly be seen in planar X-rays to allow the prediction of the existence of a safe IA bone corridor for a 3.5-mm screw. As a result, the transverse diameter of Köhler’s teardrop in the standard AP pelvic X-ray turned out as the most predictive value. In our population, the secure IA-bone corridor was found in 100% of the pelves showing a teardrop diameter of ≥ 4 mm. Hence, the teardrop diameter can be considered a safety indicator to support the surgeon’s decision to apply a 3.5-mm screw without preoperative CT.

However, an accurate preoperative analysis using conventional X-rays and CT scans remains mandatory for planning the entire surgical intervention.

Some limitations of our study must be noted. Semiautomatic CT-based segmentation and computing the IA corridor volume are time-consuming and require expensive software applications. Therefore, the presented methodological workflow is not yet feasible for applications in daily routine clinical practice. The data presented reflect only the physiological situation of the uninjured acetabulum. Any residual dislocation associated with incomplete fracture reduction would thus strongly influence all the volumetric parameters of the IA corridor and would likely lead to an insufficient screw path. Currently, the computed scripts allow only intact pelves to be analyzed. In our study population, the differences between the left and the right acetabulum were not significant; thus, measuring the contralateral acetabulum may be indicative of the corridor existence on the fractured side. Prospectively, advanced software adaptations might enable corridor computation for arbitrary patterns of acetabular fractures and a simulation of residual malreduction. Such innovations are the focus of our future research.

## Abbreviations

3-D: Three-dimensional
AI: Lequesne’s acetabular index
ANOVA: Analysis of variance
AO: Arbeitsgemeinschaft für Osteosynthesefragen
AP: Anterior posterior
BMI: Body mass index
BVV: Basic valid vector
CT: Computed tomography
DICOM: Digital Imaging and Communication in Medicine
DWI: Heyman and Herndon depth-to-width index DWI = depth of acetabulum (*a*)/width of acetabulum (*b*) × 100
HP: Hemipelvis
IA: Infraacetabular
IAC: Infraacetabular corridor
LCE: Wiberg’s lateral center-edge angle
Max: Maximum
Min: Minimum
MRI: Magnetic resonance imaging
No.: Number (numero)
ORIF: Open reduction and internal fixation
SD: Standard deviation
SPSS: Statistical Package for the Social Sciences Software by IBM
STL: Stereolithography (File Format)
